# Radiation recall pneumonitis triggered by an immune checkpoint inhibitor following re-irradiation in a lung cancer patient: a case report

**DOI:** 10.1186/s12890-022-01846-x

**Published:** 2022-02-05

**Authors:** Xianghua Ye, Jinsong Yang, Justin Stebbing, Ling Peng

**Affiliations:** 1 Department of Radiotherapy, The First Affiliated Hospital, School of Medicine, Zhejiang University, Hangzhou, Zhejiang Province China; 2 Division of Cancer, Department of Surgery and Cancer, Imperial College London, London, UK; 3 Cancer Center, Department of Pulmonary and Critical Care Medicine, Zhejiang Provincial People’s Hospital, Affiliated People’s Hospital, Hangzhou Medical College, Hangzhou, 310014 Zhejiang Province China

**Keywords:** Radiation recall pneumonitis, Radiotherapy, Immunotherapy, NSCLC

## Abstract

**Background:**

Radiation recall pneumonitis (RRP) is unpredictable but associated with severe radiation damage in previously irradiated fields. Chemotherapy and targeted drugs have been reported to contribute to RRP. Here we report a case of a patient with non-small cell lung cancer (NSCLC) who developed RRP following administration of immune checkpoint inhibitor (ICI) 18 months after the end of re-irradiation.

**Case presentation:**

A 69-year-old man received adjuvant chemoradiotherapy post-operatively. He underwent thoracic re-irradiation for oligometastatic NSCLC. On second recurrence, pembrolizumab combined with nab-paclitaxel were administered. After six months, he developed symptoms of persistent cough and dyspnea, with consistent pneumonitis on CT images. The clinical time frame and significant radiographic evidence raised suspicion for RRP. Symptoms resolved after steroids.

**Conclusions:**

RRP is a rare occurrence. Patients undergoing immunotherapy after prior irradiation may be at increased risk of this rare radiation pneumonitis.

## Background

Pneumonitis may be a potential result of both thoracic radiation and ICI, particularly treatment with programmed death-1 (PD-1) or programmed death ligand-1 (PDL-1) inhibitors [1]. The differential diagnosis between radiation pneumonitis and checkpoint inhibitor-induced pneumonitis (CIP) is of importance for patients receiving both treatments. RRP is described as a delayed inflammatory reaction occurring within irradiated tissues. RRP is rare but is reported to be triggered by chemotherapy agents such as anthracyclines, taxanes, and gemcitabine [2, 3], and targeted therapies such as erlotinib, osimertinib and vemurafenib [4, 5]. During the COVID-19 era, RRP has been reported with vaccination [6]. RRP induced by ICI has been reported, but it is uncommon in patients receiving ICI with a pre-irradiation history.

Here, we described a male patient diagnosed with primary lung squamous carcinoma who received surgery. Post-operative chemotherapy and radiotherapy were given as adjuvant therapy. On recurrence of lymph nodes 3 years post-surgery, re-irradiation was administered to mediastinal lymph nodes and chemotherapy was given. On second recurrence 4 years after surgery, systemic therapy with pembolizumab and nab-paclitaxel was given. After 6 months following initiation of pembrolizumab and nab-paclitaxel, he experienced RRP occurring 18 months after the end of re-irradiation. Steroid therapy resulted in alleviation of symptoms in 3 days and radiological improvement after one month.

## Case presentation

**Fig. 1:**
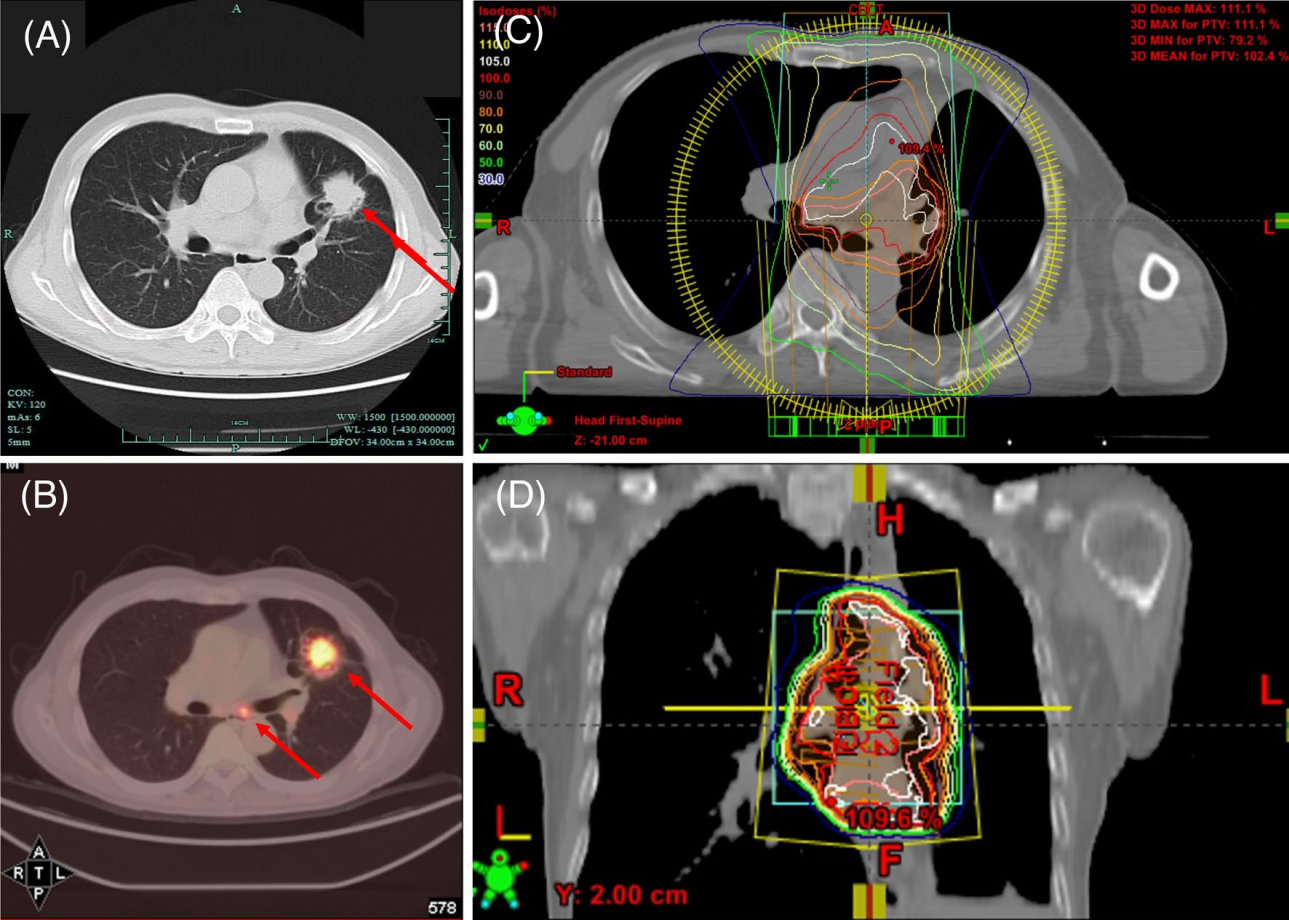
**A** CT scan of lung window captured on August 6th, 2016; **B** PET-CT captured on August 8th, 2016; **C**, **D** Radiation dosimetry post-operation. Significant abnormal findings noted (arrow)

A 64-year-old Chinese male smoker referred to The First Affiliated Hospital of Zhejiang University in August 2016 due to a one-month history of cough. His chest CT scan revealed a 3 cm × 2.5 cm mass in the left upper lobe and mediastinal lymph node enlargement (Fig. 1A). A PET-CT indicated the mass with elevated SUV and ipsilateral mediastinal lymph nodes (Fig. 1B). Past and family histories were unremarkable. Due to the staging of the disease, he was considered to be a candidate for curative surgical excision. On August 11th, 2016, this patient underwent lobectomy of left upper lobe and mediastinal lymph node dissection. Histology returned with a poorly differentiated squamous carcinoma with involvement of elastic membrane and vascular tumor embolus. The surgical margins were negative. Mediastinal lymph nodes of station 7 were metastatic with pathological stating of pT2N2M0, IIIA. Sequential chemoradiotherapy with gemcitabine/cisplatin and radiotherapy 50 Gy in 25 fractions was started 1 month after the lobectomy using Intensity-modulated radiation therapy (IMRT). Clinical target volume (CTV) includes tumor area, 4L, 5, 7 and 10L lymph node stations (Fig. 1C, D). After adjuvant chemoradiotherapy, this patient underwent routine follow-up regularly. No radiation pneumonitis was observed.

**Fig. 2:**
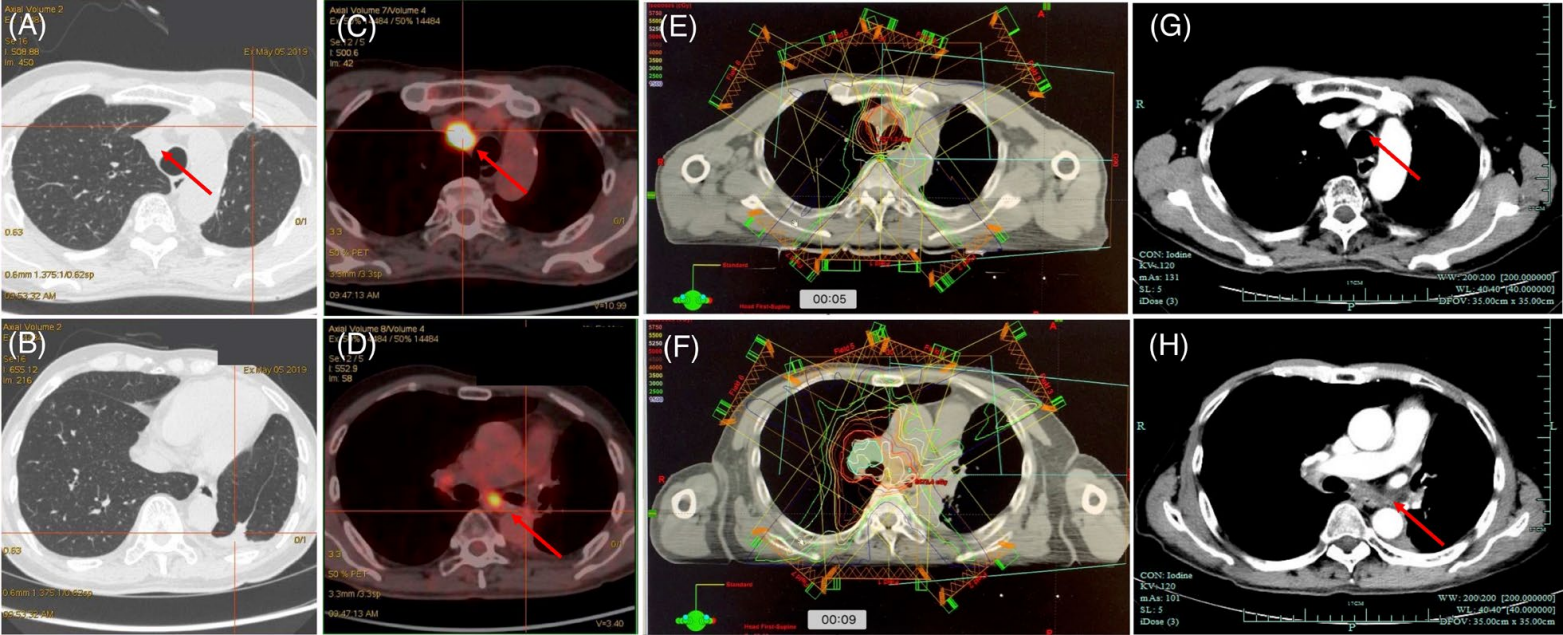
**A**–**D** PET-CT scan capture on May 5th, 2019. **E**, **F** Radiation dosimetry of re-irradiation; **G**, **H** CT lung and contrast window capture on October 29th, 2019 (after re-irradiation). Significant abnormal findings noted (arrow)

On May 2019, a chest CT scan revealed lymph node enlargement. A second PET-CT scan confirmed elevated SUV uptake of 2L, 7 stations and right hilar lymph nodes (Fig. 2A, C, D). The patient was seen in multidisciplinary follow up, and a recommendation was made for first-line chemotherapy followed by re-irradiation, which would be optimal for local-recurrence of the disease. Nab-paclitaxel with carboplatin were given for four cycles. Then, re-irradiation was given with 54 Gy/27F including 2R, 7, 10R lymph node stations from September 17th to November 14th 2019, using three-dimensional conformal radiation therapy (3D CRT) (Fig. 2E, F). The lymph nodes are shown on the follow-up CT scan one month after the end of re-irradiation (Fig. 2G, H). He tolerated well with radiotherapy and there were no symptoms. CT findings revealed no signs of radiation pneumonitis.

**Fig. 3:**
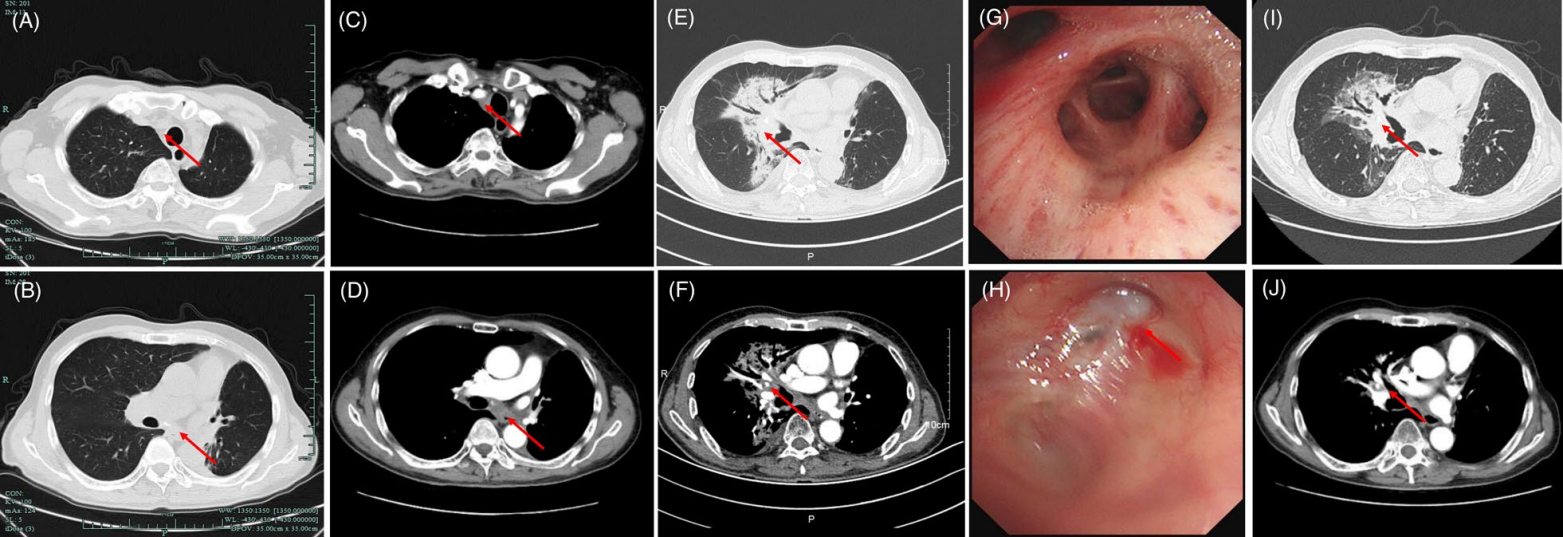
CT lung window (**A**, **B**) and contrast window (**C**, **D**) before pembrolizumab plus nab-paclitaxel, captured on November 5th, 2020; **E**, **F** CT lung and contrast window on May 1st, 2021; **G** The mucosa of right middle bronchus: is normal and the bronchus is clear. **H** The mucosa of the left upper lobe is post-surgery appearance and the bronchus is clear. **I**, **J** CT lung and contrast window captured on June 5th, 2021 (one month after steroids). Significant abnormal findings noted (arrow)

On November 2020, a CT scan revealed enlargement of lymph nodes compared with the best response to re-irradiation captured on November 2019 (Fig. 3A–D). The diagnosis of second recurrence was made. After a second multidisciplinary discussion, pembrolizumab 200 mg plus nab-paclitaxel 200 mg/m^2^ every 3 weeks were given in combination with stable disease.

**Fig. 4:**
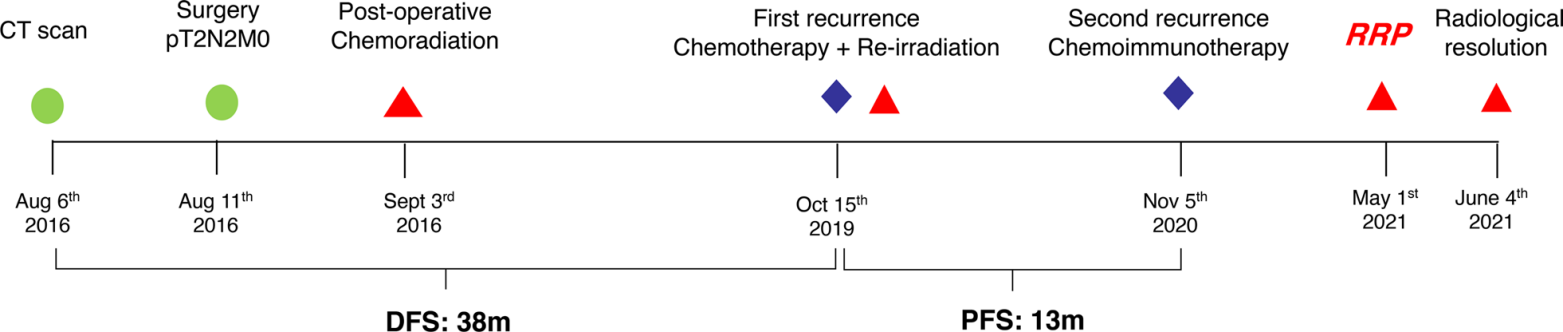
Timeline of patient history

On April 20th, 2021, he complained of persistent, nonproductive cough and dyspnea, with no fever. CT images on May 1st, 2021 indicated patchy consolidation located in the previously re-irradiated area (Fig. 3E, F). Bronchoscopy revealed no abnormalities (Fig. 3G, H). No evidence of infection and cancer cells was found from bronchoalveolar lavage fluid (BALF). Blood tests were negative for tumor marker. Cytokine levels were assessed including interleukin-2 (IL-2), IL-1, IL-6, IL-8, IL-17, interferon gamma (IFN-γ); all of which were within normal range. Steroids were given using 40 mg methylprednisolone intravenously q12h for 3 days. Concurrent moxifloxacin was given due to the potential of overlapping presentation and infection. The symptoms quickly resolved in 3 days. Steroids were tapered gradually in 6 weeks. A repeated CT scan on June 5th, 2021 revealed significant improvement of RRP (Fig. 3I, J). The timeline of his diagnosis, treatment and onset of RRP are shown in Fig. 4. At the time of writing this report, the patient is still on active follow-up.

## Discussion and conclusions

Radiation is a potent inflammation inducer. Radiation induces immunogenic cell death, which results in release of damage-associated molecular pattern (DAMP) markers [7]. Being a generator of danger signals, radiation also notifies the immune system to potential damage. Radiation has immunomodulatory effects, and the reaction of the immune system is crucial to the clinical response to radiation therapy. Inflammatory reactions are orchestrated by innate immune cells and the adaptive immune system.

With the wide application of ICIs, it is rational to explore the capacity of radiation to enhance ICI efficacy and to investigate whether combination therapy could bring benefit to patients with cancer. The overlapping pulmonary toxicity induced by thoracic RT and ICI is an important issue. Concurrent treatment with ICI and conventional therapies may also result in higher rates of pneumonitis. The safety of combination of radiotherapy and immunotherapy has been evaluated in a variety of clinical trials and real-world data. In a phase III randomized trial investigating durvalumab after concurrent chemoradiotherapy (CCRT) in stage III NSCLC, the pneumonitis rate was reported as 34%, compared to 25% in placebo arm [8]. Pneumonitis was the most frequent adverse event (AE) leading to drug discontinuation (4.8% in the durvalumab group and in 2.6% of those in the placebo group). In a real-world setting, NSCLC patients previously treated with radiotherapy had a higher risk for CIP [9]. Grade ≥ 2 CIP is an independent prognostic factor for shorter survival in NSCLC patients. On the other hand, ICI could also increase the risk for radiation-induced pneumonitis.

The differentiation of RRP and CIP is challenging in clinical setting. The clinical presentation of RRP was different from common radiation pneumonitis and RRP induced by cytotoxic/targeted drugs. RRP and CIP could both present with similar images on CT scans, and they usually respond well to steroid therapy. While the images of ICI-induced RRP represent the target volume of irradiated fields and are more focal than traditional CIP, this helps clinicians to reach a diagnosis. Radiological features of ICI-induced RRP include ground-glass or consolidative opacities confined to prior radiation field [10]. The most common radiographic patten of RRP is cryptogenic organizing pneumonia (COP) [11]. Artificial intelligence and radiomics have also emerged as promising tools to interrogate cancer images before, during and after treatment [12].

RRP is an unpredictable event, whose mechanism is not fully understood. There are some explanations postulated [11] including changes in the function of stem cells in the irradiated field versus idiosyncratic drug hypersensitivity reactions [13]. Other mechanisms as drug hypersensitivity, vascular injury or inflammatory cascades have also been suggested [14]. While the exact mechanism of RRP induced by ICI is somewhat different from conventional chemotherapy or targeted drugs, the signaling pathways involved in immunotherapy-induced RRP are thought to include cGMP-AMP synthase (cGAS)-stimulator of interferon genes (STING), nuclear factor kappa-light-chain-enhancer of activated B cells (NF-kB), reactive oxygen species/reactive nitrogen species (ROS/RNS), extracellular regulated protein kinases (Erk) and phosphatidylinositol 3-kinase (PI3K) [11].

RRP manifests as acute inflammation lymphocytes infiltration, which can occur weeks, months, or even years after radiotherapy in response to treatment. RRP is a delayed radiation-induced lung toxicity, with long interval between the administration of ICI and the onset of RRP being observed. With conventional agents, the median time of onset of RRP after the end of radiation therapy is 95 days for chemotherapy [2] and 124 days for targeted therapy [15], although onset of 2 years after radiation therapy has been reported with ICIs [16]. In our case, the onset of symptoms developed 18 months after the end of re-irradiation. The published cases of ICI-induced RRP are listed in Table 1, and the time interval of RRP to radiotherapy varies widely.

**Table 1:** RRP Cases reported

No	Author	Year	Country	Sex	Age	Malignancy	ICI	Onset time from RT
1	Current paper	2021	China	Male	69	NSCLC	Pembrolizumab	18 months
2	Riviere [24]	2021	United States	Male	64	NSCLC	Nivolumab	4.5 years
				Male	67	Bladder	Ipilimumab-Pembrolizumab	6 months
				Male	52	SCLC	Nivolumab-Ipililumab	7 months
3	De Giglio [25]	2021	Italy	Male	53	RCC	Nivolumab	10 months
4	Itamura [26]	2020	Japan	NR	NR	NSCLC	Pembrolizumab	7 months
5	Chen [27]	2020	China	Male	64	NSCLC	Camrelizumab	2 years
6	Deutsch [23]	2020	France	NR	NR	NSCLC	Nivolumab	NR
7	Wang [28]	2020	China	Male	52	SCLC	Pembrolizumab	6 months
8	McGovern [29]	2019	United States	Male	82	NSCLC	Pembrolizumab	14 months
9	Nakamura [30]	2019	Japan	Female	69	RCC	Nivolumab	9 months
10	Shibaki [16]	2017	Japan	Male	68	NSCLC	Nivolumab	2 years
				Male	55	NSCLC	Nivolumab	6 months

*RT* radiotherapy; *NSCLC* non-small cell lung cancer; *SCLC* small cell lung cancer; *RCC* renal cell carcinoma; *NR* not reported

The risk factor for RRP have not been determined, but several factors might contribute [17]: (i) Radiotherapy. The dose, fractionation, target volume, radiation technique could affect the toxicity of radiation [11]. As radiation pneumonitis is closely related to dosimetric factors, close monitoring and attention shall be given to patients receiving combination therapy. In our case report, this patient received operation upfront, followed by chemoradiation [18, 19]. In patients receiving re-irradiation, dose-volume variables from re-RT plan and factors from initial-RT are predictive factors for severe radiation pneumonitis [20]. (ii) Drugs used. Although chemotherapy, targeted drugs and ICIs can all trigger RRP, the ICI-induced RRP is different, in terms of a long interval and duration of response. In our case, this patient also received nab-paclitaxel with pembrolizumab. As a taxane is one of the drugs potentially contributing to RRP, the role of nab-paclitaxel in the development of RRP cannot be ruled out. However, as the dose and density of nab-paclitaxel was standard, we suspect the main drug in responsible for RRP here was pembrolizumab. Although previously reported, it was concluded that the pneumonitis was RRP which is a rare inflammatory reaction in the previously irradiated lung field was due to administration of this ICI. The incidences of CIP induced by anti-PD-1 antibody are higher compared to anto-PD-L1 antibody, especially grade 3–4 pneumonitis [21]. Therefore, RRP is more likely to be triggered by PD-1 inhibitor than PD-L1 inhibitor. (iii) Tumor location, molecular features and type. Whether PD-L1 status will affect the toxicity of radiation in combination with ICI remains unknown. In this patient, PD-L1 status was undermined due to the fact that his tumor sample was in 2016, when the diagnostic kit was not available in China and the achieved sample was not suitable for PD-L1 test after 5 years. Other factors as TMB (tumor mutation burden), baseline immune status, MSI status, and other peripheral blood biomarkers might also influence the onset of RRP.

Interestingly, a systematic review found that the occurrence of immune-related adverse events (irAEs) was significantly associated with a better ICI efficacy in patients with cancer, particularly endocrine, dermatological, and low-grade irAEs [22]. RRP may reflect a beneficial immune activation and constitute a predictive biomarker for long-term efficacy [23].

In this report, we describe a NSCLC patient of RRP triggered by pembrolizumab 18 months after the end of re-irradiation. RRP induced by ICIs is a unique pattern of radiation-related toxicity. Clinicians should be aware of the pulmonary toxicity even years after radiation, especially in the era of immunotherapy.

## Data Availability

The datasets used and/or analysed during the current study are available from the corresponding author on reasonable request.

## Abbreviations

RRP: Radiation recall pneumonitis
NSCLC: Non-small cell lung cancer
ICI: Immune checkpoint inhibitor
PD-1: Programmed death-1
CIP: Checkpoint inhibitor-induced pneumonitis
CTV: Clinical target volume
3D-CRT: Three-dimensional conformal radiation therapy
IFN-γ: Interferon gamma
DAMP: Damage-associated molecular pattern
CCRT: Concurrent chemoradiotherapy
AE: Adverse event
COP: Cryptogenic organizing pneumonia
cGAS: CGMP–AMP synthase
STING: CGAS-stimulator of interferon genes
NF-kB: Nuclear factor kappa-light-chain-enhancer of activated B cells
ROS: Reactive oxygen species
RNS: Reactive nitrogen species
Erk: Extracellular regulated protein kinases
PI3K: Phosphatidylinositol 3-kinase
irAE: Immune-related adverse events
